# Effect of a Collar and Harness on Intraocular Pressure and Respiration Rate of Brachycephalic and Dolichocephalic Dogs

**DOI:** 10.1002/vms3.70384

**Published:** 2025-04-28

**Authors:** Megan E. Bailey, Melissa J. Packer, Alison P. Wills

**Affiliations:** ^1^ Department of Animal and Agriculture Hartpury University Gloucestershire UK; ^2^ Farm Office, Hopsford Hall Farm Shilton Coventry UK

**Keywords:** brachycephalic dogs, collar, harness, intra‐ocular pressure, respiratory rate

## Abstract

**Background:**

Dogs are a popular pet in many countries, and for them to gain appropriate exercise, many owners opt to walk them on a leash. Despite health and welfare concerns, brachycephalic breeds remain common as pets, with limited research existing that investigates the best restraint type for these animals.

**Objectives:**

This study aimed to test the effect of a collar and harness during stationary and exercise conditions on the intra‐ocular pressure (IOP) and respiration rate (RR) of brachycephalic and dolichocephalic dogs.

**Methods:**

A total of 24 healthy dogs, both brachycephalic and dolichocephalic, were recruited for the study and underwent stationary and exercise conditions in two restraint types in a within‐between‐subjects design. IOP was measured by rebound tonometry, and RR was measured using clinical and visual methods by the same experimenter.

**Results:**

Just wearing a collar in a stationary condition increased IOP in brachycephalic dogs (*p* < 0.05) but not in dolichocephalic dogs (*p* > 0.05). Exercising in a collar increased IOP for both groups of dogs (*p* < 0.05), whereas exercising in a harness did not affect IOP for either group (*p* > 0.05). RR increased in exercise conditions for both restraint types in brachycephalic dogs (*p* < 0.05), with no difference between collar and harness (*p* > 0.05).

**Conclusions:**

Data suggest that collars may elevate IOP during exercise for all dogs and also during stationary conditions for brachycephalic breeds. Owners need to be conscious of the most appropriate restraint for their dog to avoid deleterious effects on IOP and RR.

## Graphical abstract

Collars and harnesses are common restraint types for dogs. Using a collar for brachycephalic breeds causes increases in intraocular pressure (IOP), which may be detrimental to the health and welfare of these dogs. Exercising in a harness does not increase IOP in dogs in the same way as a collar.

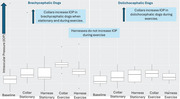

## 1 Introduction

Domestic dogs are popular pets worldwide, with an estimated 13 million dogs kept as pets in the United Kingdom (PFMA 2022) and 69 million households reporting dog ownership in the United States (Statistica 2022). Exercise is essential for canine wellbeing, and as such, dog walking is a common activity in many countries. UK legislation requires dogs to be under control in public spaces; hence, using a leash is commonplace for safety reasons (UK Government 2023). Leash pulling frequently occurs during walks (Shih et al. 2021; Walthers et al. 2019), with a survey finding 82.7% of owners reported their dogs pulling on leashes (Townsend et al. 2021). The favoured restraint for dogs is a lead attached to a neck collar (Grainger et al. 2016). However, collars are thought to localise leash pressures, damaging a dog's trachea, larynx, neck and eyes (Hunter et al. 2019; Ogburn et al. 1998). Harnesses are considered to provide better welfare, but they may also cause adverse effects such as increased pressure on the thoracic inlet or altered kinematics (Shih et al. 2021; Grainger et al. 2016; Knights and Williams 2021)

There is limited research on the impacts of collars and harnesses, despite their widespread use. Studies have shown collars to produce higher contact pressures (4.58 N/cm^2^) than those produced by ill‐fitting saddles on horses (3.8 N/cm^2^) (Hunter et al. 2019; von Peinen et al. 2010) raising welfare concerns. Moreover, the lowest collar pressure value recorded in dogs (83 kPa) was significantly higher than levels found to cause tissue damage and necrosis in humans (4.3 kPa) (Carter et al. 2020). Neck collars have been associated with respiratory issues such as tracheal collapse, which is commonly observed in small brachycephalic dog breeds like Pugs and Chihuahuas (UK Government 2014; Tappin 2016). Ventral neck pressure is also thought to compress jugular veins, obstructing ocular aqueous outflow, therefore causing increased intraocular pressure (IOP) (Pauli et al. 2006).

Research has found significant positive associations between IOP and tension applied to a collar, suggesting prioritisation of harness use may be beneficial for dogs with ocular vulnerability (Pauli et al. 2006). Brachycephalic breeds remain popular with pet owners but are predisposed to ocular issues, such as ulcers or globe proptosis (Busse 2012; Costa et al. 2021), primarily due to their prominently positioned eyes caused by conformationally shallow ocular orbits (Klein et al. 2011; Nutbrown‐Hughes 2020).

Research investigating the effects of restraint type on brachycephalic breeds is lacking, inhibiting professionals’ ability to make evidence‐based decisions and appropriately inform and educate owners. This study aimed to investigate the effect of common restraints (collar and harness) on the IOP and RR of brachycephalic and dolichocephalic dogs. It was hypothesised that collars would increase IOP and RR compared to harnesses and that this would be more marked in exercise conditions.

## 2 Material and Methods

This study was granted ethics approval from the Hartpury University Ethics Committee (REF: ETHICS2021‐32).

### 2.1 Sample Population

A sample of 24 privately owned brachycephalic and dolichocephalic dogs was acquired through purposive, opportunistic sampling via social media. Dogs had to fit specific inclusion criteria; this included being able to be leash walked, having no diagnosis of prominent ocular or respiratory abnormalities or diseases, being healthy based on clinical examination by a veterinarian, and either being classified as brachycephalic with a cephalic index greater than 60 cm or dolichocephalic with a cephalic index less than 50 cm. Exclusion criteria consisted of dogs with ocular and/or respiratory injuries or medical issues, dogs with a history of biting or aggression towards both dogs and humans and naturally anxious or overly stressed dogs as psychological stress and anxiety can increase IOP and respiratory rate (RR) (Miller and Bentley 2015) and dogs currently on medication for ocular and/or respiratory abnormalities or diseases. Dogs that had undergone ocular surgeries were excluded, as surgery purposefully decreases IOP (Komáromy et al. 2019). Likewise, dogs that had undergone respiratory surgeries were excluded, as collars are often contraindicated following surgery (Miller and Gannon 2015).

All 24 dogs met the inclusion criteria. However, three were excluded due to incomplete data, of which one also displayed a significant increase in RR with the application of a correctly fitted collar at stationary (73.33 bpm) from an established baseline RR (18.00 bpm) and thus was removed from the sample population for ethical reasons. A further dog displayed unusually high IOP values along with signs of possible ocular pathology. Therefore, for ethical reasons, data from this dog were also excluded. The remaining 20 dogs of multiple breeds encompassed 8 castrated males, 4 intact males, 7 unneutered females and one neutered female, with a mean age of 4.5 ± 2.97 years (range 8 months–8 years) and a mean body condition score (BCS) of 6.6 ± 1.23 (range 5–8). These 20 dogs were categorised into 10 brachycephalic dogs, which included 2 intact males, 4 unneutered females, 3 castrated males and 1 neutered female; mean age, 3.95 ± 3.38; mean BCS, 7.05 ± 1.01 and 10 dolichocephalic dogs, which included two intact males, three unneutered females and five castrated males; mean age, 4.98 ± 2.57; mean BCS, 6.1 ± 1.29.

### 2.2 Equipment

#### 2.2.1 Measurement of IOP and RR

Canine IOP was measured by rebound tonometry using an iCare TonoVet tonometer (iCare TA01, Tiolat Oy, Inc. Helsinki, Finland) with an accuracy of ±2 (5–30 mmHg), and 10% (30–80 mmHg), as it is well established and non‐invasive. The technique used followed manufacturer guidance (iCare TONOVET, 2015) and resembled existing research (Broadwater et al. 2008) (e.g., 90° and 4–8 mm from the central cornea). Further, the tonometer measured IOP in mmHg and was calibrated for canine use, with the display screen presenting a ‘do’ indication (iCare TONOVET, 2015). IOP was measured under the five conditions, where both eyes were measured, in order of convenience each time. An average IOP using the left and right eye results was determined for each dog, consistent with other work (Pauli et al. 2006, Williams and Gimson 2020). Some dogs allowed IOP to be measured with encouragement from the owner; however, the majority (75%) required some minimal manual restraint from the owner. Where necessary, owners were informed of correct handling or restraint protocols. The body position of IOP measurements was consistent for each dog throughout their IOP measurements, as this has been shown to affect IOP values (Broadwater et al. 2008). RR was measured in breaths per minute (bpm) (15 s, multiplied by 4) by the same individual, using both visual and/or contact clinical examination methods depending on the situation and comfort of the dog.

#### 2.2.2 Study Restraints (Collar and Harness)

Flat‐neck collars and back‐connection harnesses were used, as these were found to be the most popular restraints used by UK dog owners (Townsend et al. 2021). Restraints were from the brand RUFFWEAR; all five harnesses were RUFFWEAR Hi & Light lightweight dog harnesses (extra extra extra small to large/extra‐large), and the three collars were RUFFWEAR Front Range dog collars (small to large) (RUFFWEAR, Oregon, USA). Both restraints were correctly sized to the individual dog and fitted according to manufacturer guidance and methods described in similar studies (Hunter et al. 2019; Carter et al. 2020). To represent most UK leash dog walks, a standard 1‐m lead was used consistent with previous research (Grainger et al. 2016).

### 2.3 Experimental Protocol

The research was conducted at The Sports Horses Centre, Freeboard Lane, Ryton on Dunsmore, Rugby, CV8 3EQ. Dogs underwent a brief health and suitability check from an experienced veterinarian, where the dogs BCS was determined. A 9‐point scale was utilised as previously described (Shih et al. 2021; Shih et al. 2020). Additionally, owners confirmed their dog's health status and suitability against the inclusion and exclusion criteria.

Dogs were allowed to habituate to the environment, equipment and handler of the study. A 15‐min washout period elapsed to aid habituation and to allow RR and IOP values to return to normal in the event they were raised due to initial excitement, nervousness or stress possibly caused by transportation and unfamiliar people and environments. During the washout period, dogs were enclosed in a safe area with no restraints under constant supervision. All present individuals were instructed not to manually restrain the dog unless necessary during this time. To minimise the risk of causing increased stress, owners of more nervous dogs were asked to stay within proximity of their dog throughout the study, being within eyesight where appropriate.

Following the initial washout period, dogs underwent the five study conditions, which are outlined below. During data collection, the order allocation of the collar and harness and stationary and exercise conditions were randomised for each individual dog to reduce order effects.

1. Baseline: Baseline values of IOP and RR were taken when stationary, where the dog had no restraints, instead being handled and restrained appropriately with minimal intervention whilst IOP and RR were measured and recorded.
2. Collar whilst stationary: This consisted of dogs wearing a collar whilst stationary, where dogs were restrained by the handler, keeping the lead (attached to the collar) taut for 10 seconds (Pauli et al. 2006). The IOP and RR were then immediately measured and recorded.
3. Harness whilst stationary: Followed the exact methodology of the ‘collar whilst stationary condition’ but instead incorporated the dogs wearing a harness instead.
4. Collar with exercise: Dogs were leash walked on a loose lead by the handler on the collar restraint along a standardised route/exercise routine (dogs walked back and forth ten times along an enclosed 43 m long grass area). The handler was briefed to try and keep their walking speed consistent. The dogs' IOP and RR measurements were then immediately acquired and recorded.
5. Harness with exercise: Followed the exact methodology of the ‘collar with exercise condition’, but instead the dog was fitted with a harness rather than a collar.

Between all five conditions, the dogs were placed in 10‐min rest periods, during which they were kept under the same circumstances as described for the initial 15‐min washout period. These 10‐min rest periods were to allow IOP and RR values to return to baseline and reduce any stress potentially caused by equipment, handler and procedure interactions. Additionally, one handler was used throughout to standardise handler effects such as sex, voice tone and personality, which may influence dogs’ behaviour, stress and leash mannerisms (Shih et al. 2021; Shih et al. 2020).

### 2.4 Statistical Analysis

All statistical analyses were performed in R (version 4.2.1), packages: WRS and WRS2. Data were found to be non‐parametric; therefore, two separate robust two‐way mixed ANOVAs with bootstrapping were conducted to test for the effect of condition, dog type (brachycephalic or dolichocephalic) and interactions on IOP and RR. As a significant effect of condition was identified for both IOP and RR in the two‐way mixed ANOVAs, robust post hoc analyses were performed. For further analysis of the within‐subjects variable, the function bwbmcp was used. This function reports *p* values and critical *p* values. For these results, the null hypothesis was rejected at the *α* = 0.05 level if the *p* value was less than or equal to the critical *p* value. Data are reported for the within‐subjects variable split by the between‐subjects variable (dog type) as the function bwbmcp gives the option to run the post hoc test for both pooled and split data.

## 3 Results

### 3.1 Intraocular Pressure

**FIGURE 1:**
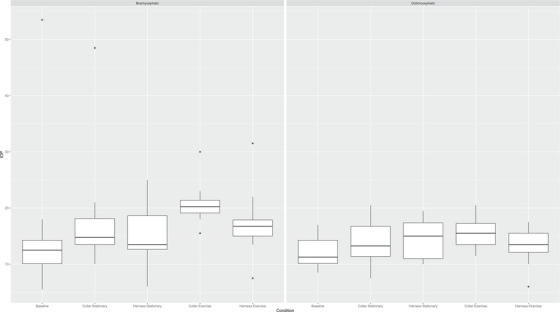
IOP values for brachycephalic and dolichocephalic dogs under five restraint and exercise conditions.

All IOP data are the mean of the left and right eye IOP data for each dog. There was no significant main effect of dog type (brachycephalic or dolichocephalic) on IOP (*p* = 0.2270). There was a significant main effect of condition (restraint and exercise) on IOP (*p* = 0.0055; Figure 1). There was no significant main interaction effect (*p* = 0.1255).

When examining brachycephalic dogs only, the mean stationary collar IOP (18.40 ± 11.03; *p* = 0.0017) and the mean exercise collar IOP (20.80 ± 3.82; *p* = 0.0006) were both significantly higher than the mean baseline IOP (15.93 ± 13.62). The mean exercise collar IOP (20.80 ± 3.82; *p* = 0.0048) was significantly higher than the mean stationary harness IOP (15.50 ± 5.82). All other pairwise comparisons were non‐significant (*p* > 0.05).

When examining dolichocephalic dogs only, the mean exercise collar IOP (15.50 ± 2.82; *p* = 0.0024) was significantly higher than the mean baseline IOP (12.10 ± 2.79). All other pairwise comparisons were non‐significant (*p* > 0.05).

### 3.2 Respiratory Rate

**FIGURE 2:**
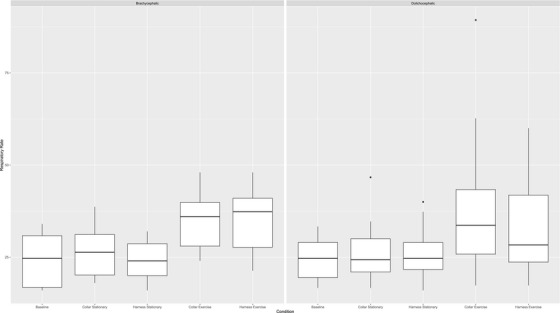
RR values for brachycephalic and dolichocephalic dogs under five restraint and exercise conditions.

There was no significant main effect of dog type (brachycephalic or dolichocephalic) on RR (*p* = 0.7585). There was a significant main effect of condition (restraint and exercise) on RR (*p* = 0.0130; Figure 2). There was no significant main interaction effect (*p* = 0.2445).

When examining brachycephalic dogs only, the stationary collar RR (26.60 ± 7.20; *p* = 0.0092), the exercise collar RR (34.93 ± 8.12; *p* = 0.0062) and the exercise harness RR (35.40 ± 8.86; *p* = 0.0003) were all significantly higher than the baseline RR (24.07 ± 7.26). The exercise harness RR (35.40 ± 8.86; *p* = 0.0002) was significantly higher than the stationary collar RR (26.60 ± 7.20). Both the exercise collar RR (34.93 ± 8.12; *p* = 0.0015) and the exercise harness RR (35.40 ± 8.86; *p* = 0.0022) were significantly higher than the stationary harness RR (24.13 ± 5.60). All other pairwise comparisons were non‐significant (*p* > 0.05).

When examining dolichocephalic dogs only, there were no significant differences in RR between any of the conditions (*p* > 0.05).

## 4 Discussion

In the present study, collars increased IOP during exercise for all dogs and even when stationary for brachycephalic dogs. Harnesses did not increase IOP for brachycephalic or dolichocephalic dogs. For dolichocephalic dogs, exercising in a collar was the only condition that significantly increased IOP above baseline.

### 4.1 Effect of Collar and Harness on IOP

The finding of the present study, where collars caused an increase in IOP, including in stationary conditions for brachycephalic dogs, aligns with existing work where IOP significantly increased when collars were worn whilst stationary when compared to baseline values (Pauli et al. 2006). The present study and Pauli et al. (2006) both found that IOP did not increase significantly when a harness was worn for both brachycephalic and dolichocephalic dogs. This suggests that harnesses have minimal impact on IOP, where effects have been documented; this is possibly caused by dogs generally pulling harder with harnesses (Shih et al. 2021) which was supported by research that noted that higher forces are generated with a harness than a collar (Pauli et al. 2006).

The present study found no significant main effect of dog type (brachycephalic and dolichocephalic) on IOP; however, when examining the effect of condition on IOP for dog types individually, there were more significant effects of different exercise and restraint conditions on brachycephalic dogs than dolichocephalic dogs. Brachycephalic dogs have a higher vulnerability to influences on IOP due to their conformational predisposition to ocular pathologies (Cebrian et al. 2021, Foote 2020) and naturally higher IOPs (Williams and Gimson 2020). IOP has been shown to be dependent on baseline values, with higher baselines presenting greater IOP changes when exercising (Najmanova et al. 2018). The higher baseline IOP values of brachycephalic dogs may explain the lack of difference observed between brachycephalic and dolichocephalic dogs in terms of the effect of exercise and restraint. A converse explanation could be that restraints affected dolichocephalic dogs more than expected; hence, less difference between dog types was observed. This was particularly evident in the exercise collar condition where IOP significantly increased compared to baseline in dolichocephalic dogs.

### 4.2 Effect of Collar and Harness on RR

The findings of the present study indicate that restraint does affect RR in brachycephalic dogs, with elevations above baseline observed in the stationary collar, exercise collar and exercise harness conditions. Furthermore, both of the exercise conditions (collar and harness) were higher than those of the stationary harness condition. Moreover, a lack of significant difference in RR between the two restraint conditions during exercise for brachycephalic dogs suggests restraint differences may be masked by exercise effects being more notable for these breeds. In contrast, exercise and restraint conditions did not significantly affect the RR of dolichocephalic dogs. This finding potentially indicates that harnesses may cause pressure to neck structures in brachycephalic dogs, where obstruction between internal structures such as the elongated soft palate and epiglottis is common (Dupré and Heidenreich 2016; Packer et al. 2015).

Harnesses in the present study were Y‐shaped, which are considered to be non‐restrictive and cause minimal impact on the dog. Furthermore, harnesses that are considered more invasive and restrictive, such as chest strap designs, may produce greater influences on canine IOP and RR than were observed in this study. Hence, further research determining the effects of different restraint designs on dogs’ physiology would inform owners, professionals and manufacturers of appropriate restraint designs for improved dog welfare.

### 4.3 Effect of Exercise on IOP

This study found significant effects of exercise conditions on IOP for both dog types, which contradicts existing literature that has shown non‐significant or negative associations between exercise and IOP (Giudice et al. 2010). However, in the present study, exercise conditions were combined with the use of restraints, meaning disentangling the effect of exercise and restraint type is challenging. Whilst a study examining the effect of unrestrained exercise on IOP would be of benefit, this does not accurately reflect how most dogs are walked, with many dogs wearing a collar and/or harness in combination with a leash.

Some research supports the findings of this study, suggesting physical exercise increases choroidal blood flow, providing increased blood to the retina and thus increased IOP (Wylȩgała 2016). Research found significant increases in participant IOP during exercise on a bicycle ergometer and a 1.7‐fold increase in IOP above resting values post‐treadmill exercise (Najmanova et al. 2018; Najmanova et al. 2016). Literature has also demonstrated increased IOP during exercise, highlighting a positive correlation between exercise intensity and IOP (Bakke et al. 2009). A confounding variable that was not controlled in the present study is water intake, which is positively associated with IOP (Chandra et al. 2011; Moura et al. 2002). Despite water restriction being achievable in human participants, there was an ethical concern in restricting the dogs’ access to water, so dogs had free constant access to water throughout the present study for welfare reasons.

### 4.4 Relationship Between IOP and RR

In the present study, restraint types and exercise affected both IOP and RR, with brachycephalic dogs showing elevations in both IOP and RR and dolichocephalic dogs predominantly demonstrating changes in IOP and not RR. The relationship between RR and IOP has been considered in human literature but is less well‐studied in dogs. The research found that increased load produced higher IOP values, linking exercise intensity and IOP (Vera et al. 2017; Vera et al. 2020). Interestingly, the latter found breathing patterns to affect IOP, with IOP increasing when gaseous exchange was compromised. This provides additional reasoning for increased IOP values during exercise in the present study, as factors like panting and stress cause abnormal breathing patterns (Bragg et al. 2015; Keren et al. 2021) comprising oxygen supply. A natural rise in blood pressure (BP) during exercise potentially explains the positive association between exercise and IOP found, with studies demonstrating transient IOP changes to parallel changes in BP during exercise (Bakke et al. 2009; Zhao et al. 2014).

There were several limitations to the present study. This study utilised a small population of dogs that were not generalisable to the canine population as a whole; therefore, the results should be interpreted cautiously in light of this. The subjective measurement of RR via thoracic wall movements likely produced inconsistencies, which could be rectified by using more advanced technology. Panting and increased activity are also indicators of stress or excitement in dogs, influencing RR and pulling frequency and intensity when leashed (Shih et al. 2021; Bragg et al. 2015). Furthermore, stress caused by the lack of habituating dogs to the tonometer and IOP examination may have confounded IOP measurements (Keren et al. 2021). This research only assessed the immediate effect of restraint type on IOP and RR and did not consider longer‐term effects. It is therefore unknown how long it takes IOP and RR to return to baseline which is an important area for future research. The study was not blinded, and while the use of a single experimenter taking the measurements reduced inter‐observer variability, there is a possibility that there was bias associated with the lack of blinding.

The present study used standard‐width collars ranging from 2 to 2.5 cm, differing from the wider collars recommended for some dolichocephalic breeds, like Poodles and Salukis. Collars with less contact surface area concentrate forces more than wider collars, potentially obstructing respiration (Hunter et al. 2019), with wider collar designs (2.8–6.2 cm) demonstrated to produce lower pressures compared to narrower collars (0.9–3.8 cm) (Carter et al. 2020). Brachycephalic breeds are predisposed to ocular abnormalities or pathologies (e.g., glaucoma) (Cebrian et al. 2021; Foote 2020) which have been associated with larger changes in IOP during exercise (Yuan et al. 2021). However, all dogs in the current study presented no historical or observable ocular disorders, but it is possible that some had sub‐clinical pathology. Activation of the sympathetic nervous system during exercise has been suggested to affect IOP in dogs (Ragusa et al. 2021). Therefore, if narrow collars are used when brachycephalic dogs experience exercise or stress, these dogs may have resultant changes in ocular physiology. Consequently, further investigation of restraints in conjunction with exercise in relation to ocular pathologies may help better inform veterinary professionals and owners to improve canine welfare.

## Author Contributions

**Megan E Bailey**: investigation, conceptualization, writing – original draft, methodology, writing – review and editing, software, formal analysis, project administration. **Melissa J Packer**: investigation, writing – review and editing, project administration. **Alison P. Wills**: conceptualization, methodology, writing – review and editing, software, formal analysis, supervision.

## Ethics Statement

Ethics approval for this study was granted by the Hartpury University Ethics Committee (REF: ETHICS2021‐32).

## Conflicts of Interest

The authors declare no conflicts of interest.

## Data Availability

The data that support the findings of this study are available on reasonable request by contacting the corresponding author.
